# The pace of a trauma resuscitation: experience matters

**DOI:** 10.1007/s00068-021-01838-2

**Published:** 2022-02-09

**Authors:** Oscar E. C. van Maarseveen, Wietske H. W. Ham, Roel L. N. Huijsmans, Luke P. H. Leenen

**Affiliations:** 1 Department of Trauma Surgery, University Medical Center Utrecht, Heidelberglaan 100, 3584 CX Utrecht, The Netherlands; 2 Emergency Department, University Medical Center Utrecht, Heidelberglaan 100, 3584 CX Utrecht, The Netherlands; 3 Institute of Nursing Studies, University of Applied Science, Heidelberglaan 7, 3584 CS Utrecht, The Netherlands

**Keywords:** Resuscitation, Trauma team, Resuscitation time, Leadership

## Abstract

**Purpose:**

Resuscitation quality and pace depend on effective team coordination, which can be facilitated by adequate leadership. Our primary aim was to assess the influence of trauma team leader experience on resuscitation pace. Second, we investigated the influence of injury severity on resuscitation pace.

**Methods:**

The trauma team leaders were identified (Staff trauma surgeon vs Fellow trauma surgeon) and classified from video analysis during a 1-week period. Resuscitations were assessed for time to the treatment plan, total resuscitation time, and procedure time. Furthermore, patient and resuscitation characteristics were assessed and compared: age, gender, Injury Severity Score, Glasgow Coma Scale < 9, and the number (and duration) of surgical procedures during initial resuscitation. Correlations between total resuscitation time, Injury Severity Score, and time to treatment plan were calculated.

**Results:**

After adjustment for the time needed for procedures, the time to treatment plan and total resuscitation time was significantly shorter in resuscitations led by a Staff trauma surgeon compared to a Fellow trauma surgeon (median 648 s (IQR 472–813) vs 852 s (IQR 694–1256); *p* 0.01 resp. median 1280 s (IQR 979–1494) vs 1535 s (IQR 1247–1864), *p* 0.04). Surgical procedures were only performed during resuscitations led by Staff trauma surgeons (4 thorax drains, 1 endotracheal intubation, 1 closed fracture reduction). Moreover, a significant negative correlation (*r*:  – 0.698, *p* < 0.01) between Injury Severity Score and resuscitation time was found.

**Conclusion:**

Experienced trauma team leaders may positively influence the pace of the resuscitation. Moreover, we found that the resuscitation pace increases when the patient is more severely injured.

## Introduction

The implementation of trauma systems and a coordinated resuscitation strategy have substantially improved the outcomes of seriously injured patients. [1] Trauma teams are one of the cornerstones of a coordinated resuscitation strategy. [2–4] The aim of a trauma team is to identify all life-threatening injuries and to provide rapid resuscitation and stabilization.

Effective team coordination is critical for the quality and pace of resuscitations and leadership can facilitate this coordination. [5] Resuscitation pace represents the speed of consecutive performed tasks and decision-making during the resuscitations. The trauma leader supervises and coordinates the team members’ activities. [6–8] The coordination of activities is of vital importance when critically injured patients are resuscitated in a team approach. During the resuscitation, multiple concurrent diagnostic and therapeutic efforts occur simultaneously.

Adequate leadership within a trauma team during a resuscitation asks for more than the correct application of the resuscitation guidelines only. Even though resuscitation guidelines provide a rational and sequential algorithmic approach, they largely focus on the technical tasks performed by individual physicians and do not address the adaptation of the complicated character of most actual resuscitations. On one hand, the complexity of resuscitation is related to the injury severity or patient characteristics, and on the other hand, this is related to the fact that in a healthcare environment, trauma resuscitations are performed by teams and not by isolated physicians. Teamwork is a complex phenomenon with many elements at play. During trauma resuscitations, for example, team members' behaviors, cognitions, and attitudes are some of the elements that interact. [9].

Previous studies have shown that non-technical skill training improves patient safety, process efficiency, and reduces medical errors by improving teamwork, including the quality of leadership of the team leader within trauma teams. [10–13] These studies, however, have evaluated resuscitation processes, such as guideline adherence and resuscitation time, primarily in a simulation setting, and did not evaluate the impact of the experience of the involved team leader. The primary aim of this study is to investigate whether the experience of a trauma team leader enhances resuscitation’s pace in real-life resuscitations. Secondary, the influence of the severity of patients’ injury on the pace of the resuscitation was investigated. Our hypothesis was that the pace of resuscitations led by a staff trauma surgeon would be faster than resuscitations led by a fellow trauma surgeon and that the pace of resuscitations would be faster when patients were more severely injured.

## Methods

### Design, data collection, and outcomes

This study is a retrospective observational study of prospectively gathered videos and data. All consecutive resuscitations of injured patients performed by a trauma team were retrospectively analyzed using videos recordings during the first week of May 2018. All included video recordings were analyzed by two researchers, with extensive experience in analyzing video recordings of trauma resuscitations. The first step in data collection was to identify trauma team leaders and categorization them into one of the following groups: trauma surgeon staff or Fellow trauma surgeons. In the Netherlands, a fellowship is the direct post-graduate period of medical specialty training as a trauma surgeon, which usually comprises 1–2 years of follow-up training. Trauma surgeon staff, however, have at least 5 years of experience as trauma surgeons. All resuscitations were assessed regarding: (A) time to treatment plan: time to the announcement of an initial treatment plan after assessment of the injured patients (T1); (B) the total resuscitation time: from patient arrival in the trauma room until patient left the resuscitation room (T2); and (C) procedural times (T3): the time from the start till the end of a surgical procedure performed during the resuscitation in the trauma room. These times were compared between the group of Fellow trauma surgeons and Staff trauma surgeons. Furthermore, patient characteristics were assessed: age, gender, Injury Severity Score (ISS), and Glasgow Coma Scale (GCS) < 9. Finally, team extension which was defined as the addition of an extra nurse or anesthesiologist supervisor and administrative delay was assessed.

### Setting

This study was performed at the University Medical Center Utrecht (UMC Utrecht), a level one trauma center in the Netherlands, which is incorporated in an academic teaching hospital. At the UMC Utrecht, we have a one-tier trauma team activation protocol, and the trauma team is activated when one of the predefined sets of trauma mechanisms, physiological, or anatomical criteria applies to admitted patients following injury. (Table 1).

**Table 1:** Criteria for activation of the trauma team

Physiological	Endotracheal intubation or endangered airway
	Respiratory distress
	Reduced level of consciousness
	Any episode of hypotension
Anatomical	Penetrating wounds proximal of the knee
	Amputation or degloving injuries
	Spinal injury
	Flail chest
	Pelvis fracture
	Two or more long bone fractures
Trauma mechanism	Fall of a height > 3 m
	Large deformity of motor vehicle
	Intrusion of passenger’s compartment
	Death of passenger in same vehicle
	Overturned car
	Pedestrian hit with > 25 km/h
	Ejected from the car
	Extrication time > 20 min
Other	Activation of trauma helicopter
	On request of ambulance personnel

In accordance with our institutional protocol, the trauma team in UMC Utrecht consists of a supervising trauma leader (a Staff trauma surgeon or Fellow trauma surgeon), a surgical resident, an anesthesiologist’s resident, an emergency care physician, two emergency department nurses, a neurologist, and a radiology technician; eight team members. In the case of severely injured patients, the nursing team may be extended to three and the anesthesiologist may be accompanied by a supervisor. The decision to extend the team is based on suspected injuries and interventions during resuscitation. The allocation of trauma surgeons (Staff or Fellow) is based on a duty schedule made by forehand, thus not based on the expected severity of the injury. A detailed description of trauma team composition and task allocation is described by Kreb et al. [14].

### Statistical analysis

For this study, we used descriptive analysis, using Microsoft Excel (Microsoft Corp. Released 2010. Microsoft Office Excel 2010, Version 14.0. Redmond, WA: Microsoft Corp.) and SPSS IBM Corp. Released 2012. IBM SPSS Statistics for Windows, Version 21.0. Armonk, NY: IBM Corp.) The skewness of data was determined using a Shapiro–Wilk test; if p values were 0.05 or higher, data were considered normally distributed. For outcomes described in percentages, percent deviation was calculated. Because of the relative sample size, both parametric and non-parametric data [time to the treatment plan (T1), total resuscitation time (T2), procedure time (T3), age, and ISS GCS] were presented as medians with Inter-Quartile Range (IQRs) and Mann–Whitney *U* test were used to compare the data of the two groups. We used the Chi-square test to compare categorical variables (gender, administrative delay and team extended). Spearman's correlation coefficient is often recommended for non-normally distributed data. However, several studies show that Pearson's correlation coefficient may offer substantial advantages in terms of statistical power for continuous non-normal data. [15, 16] Therefore, both, Pearson’s and Spearman’s rho correlation were used to determine whether statistical evidence for a relationship between Injury Severity Score (ISS) and total resuscitation time (T2) existed. A scatterplot including a trendline was made using Excel (Microsoft Corp. Released 2010. Microsoft Office Excel 2010, Version 14.0. Redmond, WA: Microsoft Corp.) The trend line was intended to visualize a general pattern of resuscitation time over ISS rather than creating a prediction model. The type of trend line with the highest *R*-squared value is displayed. *R*-squared has a value ranging from 0 to 1, with higher values indicating better prediction ability. In this study, we used cutoffs according to Chin et al. [17] for a qualitative value for the goodness-of-fit based on *R*-squared values. Values above 0.67 were considered substantial, values between 0.67 and 0.33 were considered moderate, values between 0.33 and 0.19 were considered weak, and values lower than 0.19 were considered very weak. A p value of less than 0.05 was deemed statistically significant.

### Privacy and ethics

After a waiver for approval by our institutional review board was achieved, recorded videos were analyzed, and regional data from the Dutch National Trauma Database (DNTD) were used. Trauma team members were informed of video analysis of the trauma resuscitation. Thereby, in agreement with the hospital’s legal department, informed consent from patient and personnel was not required as our institution uses video registration as part of local quality audits. For security and privacy reasons, video recordings are stored on a secured server inside the hospital building and are automatically deleted after 14 days.

## Results

### Baseline characteristics

Thirty-two videos of trauma resuscitations were included and analyzed. All provided data were normally distributed (*p* > 0.05), except for the ISS. (*p* < 0.05) (Table 2). Of the 32 analyzed resuscitations, 14 resuscitations were led by a Fellow trauma surgeon, and 18 were led by a Staff trauma surgeon. Significantly more procedures (4 thorax drains, 1 endotracheal intubation, and 1 closed fracture reduction were performed when the trauma team was led by Staff trauma surgeons compared to Fellow trauma surgeons (Staff trauma surgeons 6 procedures vs Fellow trauma surgeon 0; *p* = 0.02). No significant difference was found between ISS and GCS between the groups. (Table 2) In both groups, the teams were not extended by additional members.

**Table 2:** Baseline characteristics and resuscitation times

	Fellows	Trauma surgeons	Significance *p* value
Resuscitations, cases	14	18	NA
Number of team leaders	2	3	
Age, mean (SD)	47 (15)	53 (19)	0.41
Gender, percentage male	64	78	0.45
ISS, median, (IQR)	10 (4–26)	13 (5–21)	0.43
GCS < 9, percentage	14	6	0.40
Team extension, cases	0	0	NA
Procedures, amount	0	6	0.02*
Time to plan in seconds, median (IQR)	852 (694–1256)	719 (489–893)	0.04*
Time to plan minus procedure time in seconds, median (IQR)	852 (694–1256)	648 (472–813)	0.01*
Total resuscitation time in seconds, median (IQR)	1535 (1247–1864)	1363 (1039–1611)	0.22
Total resuscitation time minus procedure time in seconds, median (IQR)	1535 (1247–1864)	1280 (979–1494)	0.04*
Administrative delay, cases	1	1	0.92

^*^Deemed as statistical different

### The influence of experience on resuscitation’ pace

The pace of resuscitations led by a trauma surgeon was significantly faster than resuscitations led by a Fellow trauma surgeon (Table 2). The time to initial treatment plan (T1) was significantly shorter in staff trauma surgeons (Staff trauma surgeons median 719 s vs Fellow trauma surgeon 852 s; *p* = 0.04). The total resuscitation time was shorter; however, it did not reach statistical significance (Staff trauma surgeon median 1363 vs Fellow trauma surgeon 1535, *p* = 0.22). After adjustment for procedure time (as procedures were only during resuscitations led by Staff trauma surgeons), the total resuscitation time for Staff trauma surgeons was significantly shorter compared to Fellow trauma surgeons (Staff trauma surgeons median 1280 vs Fellow trauma surgeon 1535 s; *p* = 0.04).

### The influence of severity of injury on resuscitation’ pace

**Fig. 1:**
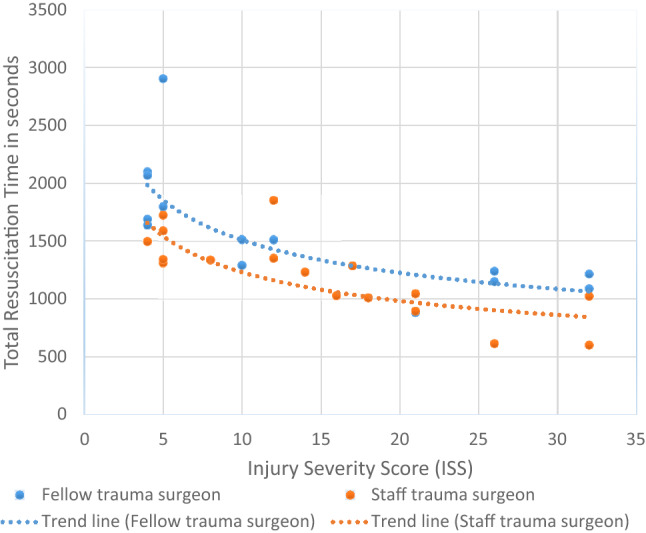
Scatterplot and trend line between injury severity score and total resuscitation time (of Trauma fellow surgeons and Staff trauma surgeons. The R-squared value trend line Staff trauma surgeon (0.59) resp. Fellow trauma surgeon (0.68)

Overall, the total resuscitation time was negatively correlated with the ISS score (*r*  – 0.698; *p* < 0.01). This was also the case for resuscitations led by both, Staff trauma surgeons and Fellows **(**Pearson’s correlation: Staff trauma surgeons: *r*  – 0.808, *p* < 0.01; Fellow trauma surgeon: *r*  – 0.712, *p* = 0.02 spearman’s correlation: Fellow trauma surgeon:  – 0.844, *p* < 0.01; staff trauma surgeons:  – 0.842, *p* < 0.01). A scatterplot with an indicative trend line is shown in Fig. 1. The goodness-of-fit of the trend lines of Staff trauma surgeons and Fellow trauma surgeons were moderate (*R*^2^ value: 0.59) and substantial (*R*^2^ value: 0.68), respectively.

## Discussion

We found a higher pace of trauma resuscitations in resuscitations that were led by experienced supervising Staff trauma team leaders compared to less-experienced team leaders (Fellow trauma surgeons). On average, the time until the treatment plan was declared, was 6 min shorter, and the total resuscitation time 6 min shorter when trauma teams were led by a Staff trauma surgeon. This difference in resuscitation pace is of clinical relevance for critically ill trauma patients requiring immediate care. Especially, for patients suffering severe hemorrhage, the enhanced resuscitation pace could be the difference between life and death. [18, 19] Second, we found a strong correlation between the severity of injury and resuscitation pace, whereas patients suffering more severe injuries were resuscitated faster. This strong correlation could be a result of situational awareness of the team (patients needing treatment fast) which leads to an expedited resuscitation. Our results are in line with the results of the study of Spanjersberg et al. [20]. In their study, the total resuscitation time was shorter in severely injured (Revised Trauma Score of 12) patients compared to less severely injured patients (Revised Trauma Score lower than 12). (34.8 versus 45.9 min). However, their results need to be interpreted with caution, as severely injured patients were resuscitated with a larger and more experienced trauma team compared to less severely injured patients.

Consistent with a previous study, [21–23], we found that more experienced trauma team leader enhances the pace of trauma resuscitations. In a before-and-after observational study performed at our hospital, we found that the introduction of a 24-h in-house attending trauma surgeon (instead of an on-call attending trauma surgeon) availability of extra experience was created within the trauma team and during initial trauma care. This resulted in a significant decrease of length of stay in the emergency department, more patients reached the ICU within an hour, and a doubling of the percentage of patients arrived in the OR within 30 min. [21] Cole et al. [22] found that in trauma resuscitations led by an experienced team leader (consultants), targets such as diagnostic imaging and hemorrhage control were significantly more likely achieved compared to trauma resuscitations that were led by less-experienced trauma team leaders (residents). Furthermore, the study of Hong et al. [23], showed that patients had a higher chance to survive when resuscitated by a team with an experienced trauma team leader. Their multivariate logistic regression analysis among 284 resuscitations revealed that a less-experienced trauma team leader (trauma surgeons with less than 2 year experience) was an important risk factor for mortality among patients with a GCS ≤ 8 [odds ratio (OR): 14.5, 95% CI 1.7–125.5, *p* = 0.015].

Although leadership can be trained, experience is still essential. In a recent randomized controlled trial regarding leadership training by Fernandez et al. [24], overall leadership scores of trauma team leaders who had received simulation leadership training were significantly higher compared to their untrained colleagues (trained leaders: mean score 10.97 SD 3.67; untrained leaders: mean score 7.27 SD 2.52; *p* = 0.02). Despite these findings, the experience remains an important cornerstone, as was demonstrated by Sarcevic et al. [25] In their study, they distinguished a directive leadership style and an empowering leadership style. A directive leadership style is preferred in resuscitations of severely injured patients with inexperienced team members. Inexperienced team members may not possess the confidence or knowledge necessary to make quick decisions. Directive leaders can help inexperienced team members by taking control of the situation. The empowering leadership style was found to be better suited during resuscitations of less severely injured patients with more experienced team members. [25] By empowering, members with more experience can make their own decisions. Therefore, the trauma team leader could focus more on decision-making and the overall plan. In the case of resuscitations of less severely injured patients, the empowering leadership style provides an educational value. Trauma leader has more time for decision-making and can discuss the situation more thoroughly with team members.

The factors that explain why resuscitation pace is faster in trauma resuscitations that are led by more experienced trauma team leaders are, to our knowledge, not studied yet. However, one could imagine that experienced trauma leaders can make decisions based on extensive experience that incorporates not only the resuscitation itself but all aspects within the complete care trajectory of trauma patients. This extensive experience enhances the pace and quality of the decision-making process during trauma resuscitation. Klein et al. [26] describe this phenomenon in detail and call it ‘recognition primed decision making’. Non-technical abilities, such as effective communication, have been shown to be critical during trauma patient resuscitation and influence resuscitation pace [5, 27]. As those non-technical skills typically develop with experience, it is reasonable to expect that resuscitations led by more experienced trauma surgeons will have a positive impact on the resuscitation pace. Furthermore, experience leads to confidence which may lead to a better overview of the process and could redirect the resuscitation process on time when needed. Finally, based on personal experience in practice, team members express higher trust in a more experienced team leader, leading to less debate during resuscitation, which is time saving.

## Strengths and limitations

The strength of this study was the use of video recordings of trauma resuscitations to analyze the pace of the resuscitation, which provides accurate documentation of the trauma resuscitation. Using video recordings allowed us to re-wind if needed, which increased the accuracy of the data collection. Our study also has some limitations. First, our study was a single-site study that enrolled a relatively small sample of 32 trauma team activations in a level one trauma center in an academic institution. The practices and policies at our institution may differ from other academic medical centers throughout and considerably vary from community-based practices. Therefore, the generalizability of our findings may be limited. Thereby, the small sample size could have introduced bias, as cofounders might be underpowered and might be overseen. There are many factors at play during trauma resuscitations, including but not limited to patient, team leader, and team member factors, not to mention interpersonal behaviors and interactions. In this explorative study, not all factors have been addressed in this study. Nevertheless, based on our results, we found that ISS could be an important cofounder when assessing the influence of the experience of the leader on resuscitation pace, as the severity of the injury of the patient seems to have an influence on resuscitation pace, as well. Furthermore, the overall severity of the injury of the resuscitated patients was medium [median 10 (IQR4-26) for Fellow trauma surgeons and 13 (IQR5-21) for Staff Trauma Surgeon; p 0.43]. The resuscitation pace is especially important for patients suffering more server injuries. The sample size of this study was too small to compare a group of the most injured patients only. However, as Fig. 1 indicates, the pace of the resuscitation led by a Fellow trauma surgeon seems, in general, to be less across all ISS scores. Therefore, it is more likely that, despite the small sample size, the results found that the experience of the trauma team leader improves resuscitation pace which is also valid for more severely injured patients. Second, patient-related outcomes were not evaluated in this explorative study. Further research is needed to investigate resuscitation pace effect on patient-related outcomes, such as length of stay, in-hospital complication, and mortality. Third, the outcome values, T1 (time to initial treatment plan), T2 (the total resuscitation time), and T3 (procedural time), might have some limitations. For the outcome time to initial treatment plan (T1) minus the procedural time (T3), bias could have potentially have been introduced, as the group who has performed most procedures (Staff trauma surgeons) may be delaying a plan to perform a procedure. However, in this study, all procedures took place after the announcement of a treatment plan and therefore have not influenced T1 minus the procedural time (T3). For the outcome total resuscitation time (T2), administrative factors might have biased the outcome. In practice, there might be some delay when the patient is leaving the trauma room when waiting for the availability of the CT scan. However, in this study only in two cases, there was a delay in transferring the patient to the CT scan. Furthermore, delays are not driven by other administrative factors (for example, bed availability, speed of transport services, etc.), while patients that do not need direct treatment are transported to another room at the ED, to prepare the shock room for new trauma resuscitations. The total resuscitation time (T1) minus procedural time might also be biased by the fact as, during procedures, plans could have been discussed and prepared for simultaneously, perhaps accounting for a seemingly quicker overall resuscitation time by the Trauma surgeon group. Fifth, the exact experience of the trauma team leader was not measured. However, the Fellows had no or only 1 year experience as trauma surgeon before the resuscitation, while staff members included in this study had at least 5 years of experience. Finally, we calculated an R-square value to assess the goodness-of-fit; however, some articles debate whether R-squared values are appropriate. [28, 29] Nevertheless, the trendline was intended to visualize a general pattern, rather than creating a prediction model. Thereby, the sample size and the fact that this is a single site would diminish the generability of such a prediction model.

### Implications for daily practice

Some considerations for daily practice, can be made. First, hospitals should consider activating a trauma team with the addition of an experienced trauma team leader in cases where it is expected that rapid resuscitation is necessary, which is typically the resuscitation of more critically injured patients. Thereby, we prefer ‘scale-down when possible’ above ‘scale-up when necessary.’ The AMC Amsterdam, a level one trauma center in the Netherlands, developed an in-hospital triage tool to downgrade the trauma team when possible to reduce over triage. The tool reduced over triage from 70% to 27%, while no undertriage was found, [30] while under triage, proportions of 42 percent are reported for two-tiered trauma call systems. [31] Furthermore, we suggest that the experienced trauma surgeon should be available from the beginning of the resuscitation, as already demonstrated in an earlier study [21]. Scaling up teams could result in undesired effects. Instead of accelerating the pace of the resuscitations as desired, the addition of a new (senior) trauma leader or other team members may interfere with the existing dynamics of the team, which could be confusing and therefore work counterproductive. Tschan et al. [32] investigated this phenomenon among cardiopulmonary resuscitations and described that the team dynamic underwent important changes each time a new team member of higher status joined the team and did not necessarily improve the performance during resuscitation. Moreover, they found that the shared responsibility of leaders leads to confusion within the team. [32] Translated to trauma resuscitations, this could be the case when experienced trauma team leader integrates during the resuscitation. Our second suggestion is to explore opportunities to improve team members’ experience; as is shown in this study, experience is an important factor for rapid resuscitation. Previous studies have shown that simulation training and video review education of actual resuscitations improved resuscitation quality and pace. [33–35]. This may imply that experience of team members, at least partly, could be based on educational and/or simulated situations and not solely on actual resuscitation-related situations. Finally, the pace of the resuscitation might be a valuable quality indicator of trauma resuscitations. For example, resuscitations could be stratified based on severity of injury and relatively slow resuscitations may be chosen for routinely evaluation during case conferences or individual educational feedback initiatives.

### Implications for research

Further research is needed to increase our understanding of leadership, experience, teamwork, and their relation to patients’ outcomes. First, consensus should be reached what leadership and experience for trauma resuscitation mean. Winston et al. [36] illustrate the complex nature of leadership. The auteurs investigated the term leadership from a sociological perspective and uncovered over 90 variables that may comprise the whole of leadership. To understand what leadership should mean during trauma resuscitation, explorative studies should analyze what factors discriminate an experienced trauma team leader discriminates from inexperienced trauma team leaders and which of these factors influences the quality and pace of trauma resuscitations. Second, research should focus on how these discriminating factors could be used to improve trauma care. For example, if some of these factors are highly trainable, such as communication skills, this should be the primary focus on training members of the trauma team. Another example could be that these factors are not trainable, but only be gained due to the experience of actual cases. In that case, it should be further defined when experienced trauma team leaders should be deployed.

## Conclusion

Experienced trauma team leaders may positively influence the pace of the resuscitation. Second, we found that the resuscitation pace increases when the patient is more severely injured.

## References

[1] Celso B, Tepas J, Langland-Orban B, Pracht E, Papa L, Lottenberg L, et al. (2006) A systematic review and meta-analysis comparing outcome of severely injured patients treated in trauma centers following the establishment of trauma systems. J Trauma [cited 2015 Jan 12];60(2):371–8; discussion 378. Available from: http://www.ncbi.nlm.nih.gov/pubmed/16508498 10.1097/01.ta.0000197916.99629.eb 16508498

[2] Driscoll PA, Vincent CA (1992). Organizing an efficient trauma team. Injury.

[3] Adedeji OA, Driscoll PA. The trauma team–a system of initial trauma care. Postgrad Med J. 1996;72(852):587–93. 10.1136/pgmj.72.852.587 PMC2398610 8977939

[4] Gondek S, Schroeder ME, Sarani B (2017). Assessment and resuscitation in trauma management. Surg Clin North Am.

[5] El-shafy IA, Delgado J, Akerman M, Bullaro F, Christopherson NAM, Prince JM (2018). Closed-loop communication improves task completion in pediatric trauma resuscitation. J Surg Educ.

[6] Hjortdahl M, Ringen AH, Naess AC, Wisborg T (2009). Leadership is the essential non-technical skill in the trauma team - Results of a qualitative study. Scand J Trauma Resusc Emerg Med..

[7] Salas E, Sims DE, Shawn BC (2005). Is there A “big five” in teamwork?. Small Gr Res.

[8] Ford K, Menchine M, Burner E, Arora S, Inaba K, Demetriades D, et al (2016) Leadership and Teamwork in Trauma and Resuscitation. West J Emerg Med 17(5):549–56. Available from: http://escholarship.org/uc/item/0jr3k73t 10.5811/westjem.2016.7.29812 PMC5017838 27625718

[9] Lacerenza CN, Marlow SL, Tannenbaum SI, Salas E (2018). Team development interventions: Evidence-based approaches for improving teamwork performance measurement in simulation-based-training view project. Am Psychol.

[10] Bassin B, Murray J, Benner C, Sikavitsas A, Santen S, Nypaver M (2014). Improvement in non-technical skills using an in-situ multidisciplinary simulationbased trauma team training (T3) curriculum. Acad Emerg Med.

[11] Gjeraa K, Moller TP, Ostergaard D (2014). Efficacy of simulation-based trauma team training of non-technical skills. A systematic review. Acta Anaesthesiol Scand.

[12] A. B, A.S. R, M.F. J, S.J. Y, W. J, S.R. L, et al. The Role of Nontechnical Skills in Simulated Trauma Resuscitation. J Surg Educ [Internet]. 2015; Available from: http://www.embase.com/search/results?subaction=viewrecord&from=export&id=L603447054 10.1016/j.jsurg.2015.01.020 25817012

[13] Westli HK, Johnsen BH, Eid J, Rasten I, Brattebø G Teamwork skills, shared mental models, and performance in simulated trauma teams: An independent group design. Scand J Trauma Resusc Emerg Med 2010;18(1):1–8. 10.1186/1757-7241-18-47 PMC2939527 20807420

[14] Tiel Groenestege-Kreb D, van Maarseveen O, Leenen L (2014). Trauma team. Br J Anaesth.

[15] Chok NS (2010) Pearson’s versus Spearman’s and Kendall’s correlation coefficients for continuous data. University of Pittsburgh

[16] Bishara AJ, Hittner JB (2012). Testing the significance of a correlation with nonnormal data: Comparison of Pearson, Spearman, transformation, and resampling approaches. Psychol Methods.

[17] Chin WW (1998). The partial least squares approach to structural equation modeling. Mod methods Bus Res.

[18] Tien HC, Spencer F, Tremblay LN, Rizoli SB, Brenneman FD (2007). Preventable deaths from hemorrhage at a Level I Canadian trauma center. J Trauma Inj Infect Crit Care.

[19] Howell GM, Peitzman AB, Nirula R, Rosengart MR, Alarcon LH, Billiar TR (2010). Delay to therapeutic interventional radiology postinjury: Time is of the essence. J Trauma Inj Infect Crit Care.

[20] Spanjersberg WR, Bergs EA, Mushkudiani N, Klimek M, Schipper IB Protocol compliance and time management in blunt trauma resuscitation. Emerg Med J 2009;26(1):23–7. Available from: http://search.ebscohost.com/login.aspx?direct=true&db=cin20&AN=105400598&site=ehost-live 10.1136/emj.2008.058073 19104091

[21] van der Vliet QMJ, van Maarseveen OEC, Smeeing DPJ, Houwert RM, van Wessem KJP, Simmermacher RKJ (2018). Severely injured patients benefit from in-house attending trauma surgeons. Injury.

[22] Cole EM, West A, Davenport R, Naganathar S, Kanzara T, Carey M (2013). Can residents be effective trauma team leaders in a major trauma centre?. Injury.

[23] Hong ZJ, Chen CJ, Chan DC, Chen TW, Yu JC, Der HS (2019). Experienced trauma team leaders save the lives of multiple-trauma patients with severe head injuries. Surg Today.

[24] Fernandez R, Rosenman ED, Olenick J, Misisco A, Brolliar SM, Chipman AK (2020). Simulation-based team leadership training improves team leadership during actual trauma resuscitations: a randomized controlled trial. Crit Care Med.

[25] Sarcevic A, Marsic I, Waterhouse LJ, Stockwell DC, Burd RS (2011). Leadership structures in emergency care settings: a study of two trauma centers. Int J Med Inform.

[26] Klein GA (1993). A recognition-primed decision (RPD) model of rapid decision making. Decis Mak Action Model methods.

[27] Pucher PH, Aggarwal R, Batrick N, Jenkins M, Darzi A (2014). Nontechnical skills performance and care processes in the management of the acute trauma patient. Surgery.

[28] Cameron AC, Windmeijer FAG (1997). An R-squared measure of goodness of fit for some common nonlinear regression models. J Econom.

[29] Onyutha, C. From R-squared to coefficient of model accuracy for assessing" goodness-of-fits", *Geosci Model Dev Discuss*, 2020;4:1–25. 10.5194/gmd-2020-51

[30] Jin PHPFK, Van Olffen TBM, Goslings JC, Luitse JSK, Ponsen KJ (2006). In-hospital downgrading of the trauma team: Validation of the Academic Medical Center downgrading criteria. Injury.

[31] Curtis K, Olivier J, Mitchell R, Cook A, Rankin T, Rana A (2011). Evaluation of a tiered trauma call system in a level 1 trauma centre. Injury.

[32] Tschan F, Semmer NK, Gautschi D, Hunziker P, Spychiger M, Marsch SU (2006). Leading to recovery: Group performance and coordinative activities in medical emergency driven groups. Hum Perform.

[33] Capella J, Smith S, Philp A, Putnam T, Gilbert C, Fry W (2010). Teamwork training improves the clinical care of trauma patients. J Surg Educ.

[34] Steinemann S, Berg B, Skinner A, Ditulio A, Anzelon K, Terada K, et al (2011) In situ, multidisciplinary, simulation-based teamwork training improves early trauma care. J Surg Educ 68(6):472–477. Available from: http://www.ncbi.nlm.nih.gov/pubmed/22000533 10.1016/j.jsurg.2011.05.009 22000533

[35] Hoyt DB, Shackford SR, Fridland PH, Mackersie RC, Hansbrough JF, Wachtel TL, et al (1998) Video recording trauma resuscitations: an effective teaching technique. J Trauma 28(4):435–40. Available from: http://www.ncbi.nlm.nih.gov/pubmed/3352005 10.1097/00005373-198804000-00003 3352005

[36] Winston B, Patterson K (2006). An integrative definition of leadership. Int J Leadersh Stud.

